# Optical data of graphene grown by chemical vapor deposition on copper foil using spectroscopic ellipsometry

**DOI:** 10.1016/j.dib.2022.108327

**Published:** 2022-06-03

**Authors:** Fahd Rajab

**Affiliations:** a Chemical Engineering Department, College of Engineering, Najran University, Saudi Arabia; b Promising Centre for Sensors and Electronic Devices (PCSED), Advanced Materials and Nano-Research Centre, Najran University, P.O. Box: 1988, Najran 11001, Saudi Arabia

**Keywords:** CVD, ellipsometry, amplitude ratio, copper foil, graphite box

## Abstract

Hexagonal, carbon-based structures were prepared on bare and treated copper foil substrates by Chemical Vapor Deposition at 1075 ^ᵒ^C using a graphite box for limiting copper reactivity with hydrocarbon gases during growth reactions. Spectroscopic ellipsometry with a digital camera was used to collect the amplitude ratio and the phase of structures various spots. The amplitude ratio of hexagonal structures on copper substrates was collected based on substrate treatment type and thickness as well as reaction zone conditions and methane flow rates. Matched amplitude ratio was obtained only for 75 µm thick acetic acid copper foil substrates inside and outside the graphite box at a low methane flow rate of 0.2 sccm. Moreover, the amplitude ratio of 75 µm bare copper foil and 75 µm electro-polished copper foil substrates at methane flow rates of 0.2 - 0.3 sccm were unmatched. Ellipsometric parameters data use potential is assessment of graphene layer thickness on different surfaces. Data shows dependence of the amplitude ratio on the surface roughness of copper foil, gas concentrations and reaction zone conditions.

## Specifications Table

**Table:** 

Subject	Materials Science
Specific subject area	Material Characterization
Type of data	Graph Figure
How the data were acquired	Synthesis of materials on copper foil substrates was carried out in a FirstNano EasyTube 2000 Chemical Vapor Deposition system at varying conditions. Ellipsometric parameters of materials on copper foil substrates were obtained using V-VASE Ellipsometer from JA Woollam Co. Data was collected at incidence angle of 70 degrees.
Data format	Data are in raw format and have been analyzed. A file with data has been uploaded.
Description of data collection	Each set was composed of 25 µm and 75 µm acetic acid treated copper foil, 75 µm bare copper foil, and 75 µm electro-polished copper foil. Two sets of copper foil substrates were used in a single CVD experiment; one within the graphite box and another outside. CVD experiments were run at methane flow rates of 0.3 sccm - 0.2 sccm. The amplitude ratio of materials on copper foil substrate was generated based on copper foil substrate thickness and methane flow rate.
Data source location	• Institution: Najran University • City/Town/Region: Najran • Country: Saudi Arabia • Latitude and longitude: 17° 29′ 17.99″ N and 44° 07′ 33.60″ E
Data accessibility	Repository name: Mendeley Data Data identification number: DOI:10.17632/cvxb9y2ymn.3 Direct URL to data: https://data.mendeley.com/datasets/cvxb9y2ymn/3 Rajab, Fahd (2022), “Amplitude ratio data of hexagonal structures prepared by Chemical Vapor Deposition on copper foil substrates”, Mendeley Data, V3, doi:10.17632/cvxb9y2ymn.3

## Value of the Data

• This data is useful because it validates a non-destructive analysis technique of a wide range of materials on different surfaces including graphene and/or graphite on copper foils.
• This data can provide insight on materials class, properties and structure, which are required by materials scientists and advanced materials product developers.
• This data can be used in further experiments to probe materials class and uniformity based on CVD hydrocarbon flow rates and surface roughness of materials due to substrate thickness and treatment methods.
• This data can be used to obtain the refractive index (n) and extinction coefficient (k), as well as film thickness by fitting data to model for films on substrates.

## 1 Data Description

**Fig. 1:**
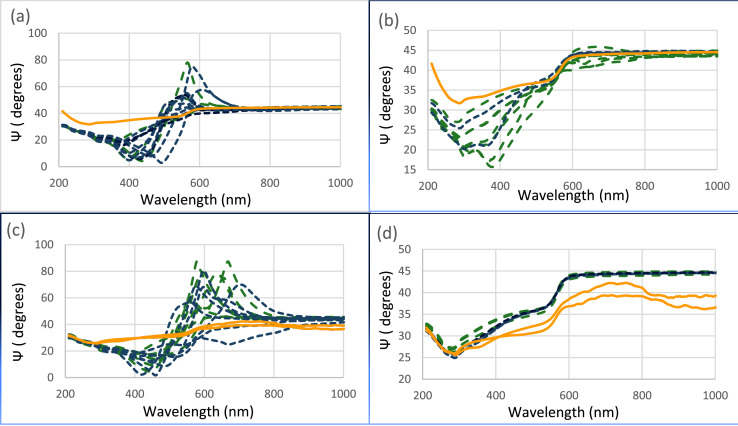
The amplitude ratio of hexagonal structures on acetic acid treated copper foil substrates within (blue) and outside (green) of graphite box as well as control substrates (yellow), at varying substrate thickness, and methane flow rate (a) 25 µm, 0.3 sccm (b) 25 µm, 0.2 sccm (c) 75 µm, 0.3 sccm and (d) 75 µm, 0.2 sccm.

This data shows optical characterization of CVD graphene's by spectroscopic ellipsometry. Amplitude ratio (Ψ) is related to the refractive index (n). When the psi peak-to-peak height is large, it indicates a large film-substrate refractive index difference and vice versa. Multiple amplitude ratio data files were collected from V-VASE Ellipsometer for various hexagonal structures on bare and treated copper foil substrates. Excel software was used to generate Fig. 1 a-d based on foil substrate thickness and gas flow. Fig. 1 shows the amplitude ratio of film within the graphite box (blue) and outside (green) on acetic-acid, treated copper foil substrate thickness of (a) and (b) 25 µm, (c) and (d) 75 µm, and methane flow rate of (a), (c) 0.3 sccm and (b), (d) 0.2 sccm as well as control samples (yellow).

**Fig. 2:**
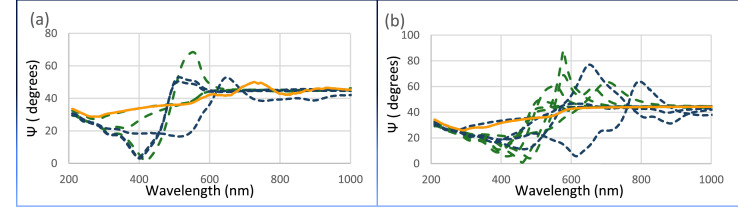
The amplitude ratio of hexagonal structures on 75 µm bare copper and 75 electro-polished copper foils substrates within (blue) and outside (green) of graphite box as well as control substrates (yellow), at methane flow rate of 0.3 sccm.

Partially matched amplitude ratio (550-1000 nm) was obtained for film on 25 µm thick acetic-acid, treated copper foil substrate at a low methane flow rate of 0.2 sccm, Fig 1b, whereas fully matched amplitude ratio (200-1000 nm) was only obtained for film on 75 µm thick acetic-acid, treated copper foil substrate at a the same low methane flow rate of 0.2 sccm, Fig. 1d, indicating a constant graphene refractive index and small graphene-film refractive index difference. When the methane flow rate was increased to 0.3 sccm, similar film on copper foil substrate showed unmatched amplitude ratio at wavelengths greater than 300 nm, Fig. 1a, c. Moreover, when film on 75 µm bare copper foil substrate and 75 µm electro-polished copper foil substrate were used at methane flow rate of 0.3 sccm, the amplitude ratio were unmatched at the entire wavelength range, Fig. 2 c, indicating a varying film refractive index and a large film-substrate refractive index difference.

## 2 Experimental Design, Materials and Methods

### 2.1 Experimental Design

Each experimental set was composed of (∼ 2 cm x 2 cm) 75 µm bare copper foil, 75 µm acetic acid copper foil, 25 µm acetic acid copper foil, and 75 µm electro-polished copper foil substrates. One set of copper foil substrates was placed within the graphite box enclosure and another set of copper foil substrates was placed outside the graphite box at methane flow rates of 0.3 sccm and 0.2 sccm. A graphite box was used to allow increase of reaction temperature from 1000 ᵒC to a temperature just below copper melting point (1075 ᵒC) to limit copper reactivity with hydrocarbons. Ellipsometric parameters of materials on copper foil substrates were collected at an incidence angle of 70 degrees [1], [2], [3]. The amplitude ratio of materials on copper foil substrate vs. wavelength was generated on separate graphs based on copper foil substrate thickness and methane flow rate [4], [5], [6], [7].

### 2.2 Materials

75 µm bare copper foil, 75 µm acetic acid, 25 µm acetic acid, and 75 µm electro-polished copper foil were acquired from CVD Materials Corporation. 99.99 % Hydrogen, Methane, Argon, Nitrogen, and Air gases were used as received.

### 2.3 Methods

Synthesis of materials on copper foil substrates was carried out in a FirstNano EasyTube 2000 Chemical Vapor Deposition system at varying conditions. For each experiment, two sets of copper foil substrates were placed within and outside of the graphite box. Each set was composed of 25 µm and 75 µm acetic acid treated copper foil, 75 µm bare copper foil, and 75 µm electro-polished copper foil substrates. The chamber was heated to 1075 °C under flow of 4900 sccm of Argon for 60 minutes at atmospheric conditions. The samples were kept at 1075 °C under flow of 150 sccm H_2_ for 5 minutes to deoxidize the catalysts. Then, 0.2-0.3 sccm (39-59 ppm) of CH4 and 35 sccm of ethylene were added for 60 min. Then, the furnace was cooled to room temperature in Ar gas to prevent oxidation of materials. Ellipsometric parameters of materials on copper foil substrates were obtained using V-VASE Ellipsometer from JA Woollam Co. at incidence angle of 70 degrees. Data of the amplitude ratio of materials on copper foil substrates was collected based on copper foil substrate thickness and methane flow rate.

## Ethics Statements

The author has complied with ethical standards.

## CRediT Author Statement

**Fahd Rajab:** Conceptualization, Data curation, Writing – original draft preparation, Visualization, Investigation, Supervision, Writing – review & editing.

## Declaration of Competing Interest

The authors declare that they have no known competing financial interests or personal relationships that could have appeared to influence the work reported in this paper.

## Data Availability

Amplitude ratio data of hexagonal structures prepared by Chemical Vapor Deposition on copper foil substrates (Original data) (Mendeley Data).
